# Antibody kinetics between birth and three months of life in healthy infants with natural exposure to Group B streptococcus: A UK cohort study

**DOI:** 10.1016/j.vaccine.2024.04.014

**Published:** 2024-05-10

**Authors:** Konstantinos Karampatsas, Tom Hall, Merryn Voysey, Clara Carreras-Abad, Madeleine Cochet, Laxmee Ramkhelawon, Elisabeth Peregrine, Nick Andrews, Paul T. Heath, Kirsty Le Doare

**Affiliations:** a Centre for Neonatal and Paediatric Infection, St. George's, University of London, London, United Kingdom; b Oxford Vaccine Group, Department of Paediatrics, University of Oxford, Oxford, United Kingdom; c Department of Obstetrics and Gynaecology, Kingston Hospital NHS Foundation Trust, London, United Kingdom; d UK Health Security Agency, London, United Kingdom; e Pathogen Immunology Group, UK Health Security Agency, Porton Down, United Kingdom; f Makerere University Johns Hopkins University, Kampala, Uganda

**Keywords:** Group B streptococcus, Maternal vaccination, Antibody kinetics, Correlate of Protection

## Abstract

**Introduction:**

Capsular polysaccharide (CPS) serotype-specific Immunoglobulin G (IgG) in cord blood has been proposed as a correlate of protection against invasive Group B Streptococcus (iGBS) disease. Although protective levels are required in infants throughout the window of vulnerability up to 3 months of age, little is known regarding the kinetics of GBS-specific IgG over this period.

**Methods:**

We enrolled 33 healthy infants born to mothers colonized with GBS. We collected cord blood and infant blood samples either at one (21–35 days), two (49–63 days), or three months of age (77–91 days). We measured GBS serotype-specific CPS IgG concentrations and calculated the decay rate using a mixed-effects model. We further explored whether the antibody kinetics were affected by common maternal and infant factors and estimated the correlation between IgG concentration at birth and one, two, and three months of age.

**Results:**

The half-life estimate of IgG concentration for homologous and non-homologous GBS serotypes in paired samples with detectable IgG levels at both time points was 27.4 (95 % CI: 23.5–32.9) days. The decay rate did not vary by maternal age (p = 0.7), ethnicity (p = 0.1), gravida (p = 0.1), gestation (p = 0.7), and infant sex (p = 0.1). Predicted IgG titres above the assay lower limit of quantification on day 30 strongly correlated with titres at birth (Spearman correlation coefficient 0.71 [95 % CI: 0.60–0.80]).

**Conclusion:**

Our results provide a basis for future investigations into the use of antibody kinetics in defining a serocorrelate of protection against late-onset iGBS disease.

## Highlights

• Point estimates of the half-life of transplacental anti-GBS IgG were 23–28 days.
• Strong correlation between IgG concentrations at birth and one month of age.
• Antibody kinetics may help in defining a correlate of protection against GBS LOD.

## 1 Introduction

Group B streptococcus (GBS) is one of the leading causes of neonatal sepsis and meningitis in most countries [1] and is associated with significant mortality and morbidity, including long-term neurodevelopmental sequelae [2]. The highest incidence of invasive GBS (iGBS) disease is in the first 90 days of life, and the condition is traditionally divided into early-onset disease (EOD, occurring in infants aged < 7 days) and late-onset disease (LOD, occurring in infants aged 7–89 days). Intrapartum antibiotic prophylaxis (IAP) for women antenatally colonized with GBS interrupts the vertical transmission of GBS to the newborn infant and decreases the incidence of EOD [3], but has no impact on LOD, GBS-related stillbirths and premature births, or disease in pregnant women [3], [4], [5]. Given the shortcomings of IAP-based prevention strategies and evidence that maternal antibodies acquired after natural exposure may protect the young infant from iGBS disease [6], vaccination in pregnancy is a promising prevention strategy [7].

The ability of maternal antibodies to protect against infant GBS disease was first reported in 1976 by Baker and Kasper in a study that showed an association between low serotype-specific capsular polysaccharide (CPS) antibody levels and a high risk of iGBS disease in newborns [8]. In most subsequent studies, low levels of CPS antibodies were found in maternal birth sera of women who had infants with iGBS disease compared with sera from women delivering infants who remained healthy [9], [10], [11], [12]. The concentrations of antibodies transferred across the placenta differ between infants and mainly depend on maternal antibody concentrations [13]. Immunoglobulin G (IgG) transfer starts early in pregnancy (10 % of maternal IgG is transferred between 17 and 22 weeks) [14] and reaches higher values towards the end of gestation as the neonatal Fc receptor (FcRn) expression on the syncytiotrophoblast increases [15], [16]. Previous studies found that IgG transfer rates vary significantly by IgG subclass [17], antigen specificity [18], and antibody glycosylation [19]. Less is known about the kinetics of naturally acquired anti-GBS CPS IgG after birth. Due to the increasing focus on defining a serocorrelate of protection derived from natural exposure to GBS to facilitate vaccine development and licensure, it is important to have accurate estimates of naturally acquired CPS antibody half-lives [20]. In addition, the antibody decay rate might be affected by epidemiological and clinical factors. Furthermore, if antibody levels measured in the first weeks of life can be used to accurately back-calculate birth titres, acute sera from LOD cases could be used in studies when no birth sera are available.

In this study, we collected data from healthy infants born to women colonized with GBS to estimate the kinetics of anti-GBS CPS IgG between birth and three months of life and to explore factors that might influence the rate of decay, focussing on maternal age, ethnicity, gravida, gestation, and infant sex.

## 2 Material and methods

The iGBS feasibility study was a prospective cohort study of pregnant women and their infants conducted in two phases [21]. The aim of the study was to test key operational aspects of a large seroepidemiological study (iGBS3) to determine the correlate of protection against the major iGBS disease-causing serotypes in infants in the UK. In the first phase, participants were recruited from five maternity units in London and South England for six months from 1 July to 31 December 2018. The study was continued for another 13 months, from 1 October 2019 to 30 November 2020, in three maternity units. The inclusion criteria were all pregnant women aged 18 or above delivering at one of the selected hospitals during the study period. Women consented to provide cord blood samples as well as a rectovaginal swab. During the second phase, two study centres also participated in the kinetics sub-study where healthy infants from women colonized with GBS were randomized to have a blood sample collected at either one (Group A: day 21–35), two (Group B: day 49–63) or three (Group C: day 77–91) months of age. Simple randomization was performed using freely available software. Group allocations were placed inside sequentially numbered opaque envelopes by a member of the research team who was not involved in recruitment and blood collection. On recruitment to the sub-study, each participant was allocated, in order of inclusion, the next available envelope, after written informed consent was obtained by the parent/legal guardian of the participating infants. Ethics approval for the study was given by the West Midlands–Solihull Research Ethics Committee on 15 June 2018 with reference number 18/WM/0147.

### 2.1 Laboratory methods

#### 2.1.1 GBS identification and serotyping

A single rectovaginal swab was obtained at any time from 35 weeks gestation up to (and including) delivery. The swab was inoculated into the selective enrichment culture medium (ECM), Todd-Hewitt broth with 10 μg colistin and 15 μg nalidixic acid (LIM broth), incubated for 6 to 24 h at 37 °C, followed by culture onto CHROMagar for 18–24 h at 37 °C with 5 % CO2 [22] Once GBS colonies were positively identified, confirmed by Matrix-Assisted Laser Desorption/Ionization Time Of Flight Mass Spectrometry (MALDI-TOF MS) and grown pure on blood agar, the serotype was determined using polymerase chain reaction (PCR) according to the methods previously reported [23], [24].

#### 2.1.2 Detection of IgG in sera

We measured serotype-specific anti-GBS CPS IgG concentrations using the GASTON-adopted multiplex immunoassay (MIA) in cord and infant sera [25]. The GASTON MIA measures anti-CPS antibodies in human serum samples specific to GBS serotypes Ia, Ib, II III, IV and V. The assay is based on Luminex technology; samples were incubated overnight with a 6-plex pool of beads, with each bead region coupled to either serotype Ia, Ib, II, III, IV, or V polysaccharide poly-L-lysine conjugates (Pfizer, Inc.). Samples were diluted to 1/500, 1/5,000, and 1/50,000. Each plate included an 11-point standard curve of multivalent vaccinee reference serum (Pfizer Inc, New York, NY, USA) serially diluted 2.5-fold, and two wells containing assay buffer acted as blank controls [26]. The next day, the plates were washed with 200 µL of phosphate-buffered saline (PBS)/Tween (1xPBS/0.05 %Tween/0.02 %/Sodium Azide, pH 7.2) using a plate washer (Tecan Hydrospeed, Tecan, Reading, UK) with a magnetic base to retain the beads. Secondary antibody was added (R-Phycoerythrin Goat Anti-Human IgG Fcy specific, Jackson Laboratories 109–115-098, Jackson ImmunoResearch Ely, UK), at 1/500 (50 µL) to each well, and the plates were incubated for 90 (±15) minutes at room temperature under constant shaking. Following the incubation, the plates were washed again, and 100 µL of wash buffer added to each well. The plates were read on a Bio-plex 200 (Bio-Rad Laboratories, Hercules, CA, USA) at high RP1 (high photomultiplier tube voltage). Data were captured as median fluorescent intensities (MFI) and converted to mcg/mL antibody concentrations using the reference standard curve and accounting for the serum dilution factor. The standardised lower limit of quantitation (LLOQ) values were published recently [27].

### 2.2 Statistical methods

The serotype-specific antibody decay rate for each infant was calculated as the change in log10 IgG between the infant blood serum and the cord serum, divided by the number of days between the two samples. A mixed effects model with infant-specific random intercepts was used on log10 concentrations at all time points to calculate the mean slope and its 95 % confidence interval (CI). The half-life of antibodies with 95 % CI was calculated as log10(0.5)/log10 transformed mean slope. We estimated the decay rate of IgG against the serotype that the infants’s mother was found to be colonized with (the homologous serotype) as well as IgG against the other five serotypes the mother was not colonized with (non-homologous serotypes). Due to the small number of participants, data from all serotypes were combined to estimate the mean half-life of IgG as the primary analysis, with serotype-specific mean half-lives presented as a Supplementary analysis. A value of half the serotype-specific LLOQ was assigned to samples below LLOQ.

Some samples were excluded from the primary analysis of half-lives following similar exclusion criteria to those proposed in previous *meta*-analyses of transplacental antibodies [28], [29]. Participants with values below the LLOQ at both time points were excluded since the half-lives cannot be estimated. Participants with increasing antibodies after birth were excluded, as such results give negative half-lives and are likely outliers. Some samples were excluded due to the technical specifications of the assay. Infants born to mothers colonized with serotype VI, against which the GASTON MIA does not measure IgG, or when the serotype identification failed, were excluded from the analysis of the decay rate against the colonizing serotype, but included when titres against non-homologous serotypes were used. To examine the influence of values below LLOQ, a sensitivity analysis of IgG against homologous and non-homologous CPS was performed using only samples with IgG titres > LLOQ at both time points. The exclusions in this sensitivity analysis also yield a dataset that would best be used to back-calculate birth tires from observed titres above the LLOQ since any results below LLOQ could only be back-calculated to the same < LLOQ level.

To calculate whether decay rates differed by time-invariant covariates such as maternal age, ethnicity, gravida, gestation, and infant sex, we used a mixed effects model, with participant-specific random intercepts, applied to the data from the combined analysis (homologous and non-homologous serotypes) and included the covariate in the model as a fixed effect, along with the covariate-by-day-interaction term. The interaction term p value was used to test if decay rates varied by time-invariant factors.

To evaluate the potential use of titres within 90 days after birth on back-calculating birth titres, a two-step analysis was done based on predicted titres at 30, 60 and 90 days. Predictions were made for all samples with birth titres above LLOQ (homologous and non-homologous serotypes) by using the slope between the observed logged titres for each individual at birth and the time point available and then interpolated or extrapolated this to get the estimated titre at 30, 60 and 90 days. Values below serotype-specific assay LLOQ after birth were given a value of half LLOQ. The first step was to assess the proportion of samples above LLOQ at birth that fell to a predicted level below LLOQ and the proportion of samples below LLOQ that raised above LLOQ at each time point. The same was done using cut-offs of 0.1 mcg/mL and 1 mcg/mL to evaluate these potential correlates of protection. Where the proportion of samples falling below LLOQ was high (above 10 %) at any time point, it was deemed not reliable to back-calculate birth titres from data at this time point. The next step was to estimate the correlations between IgG at birth and the predicted titres from all included samples at each time point. This was done using Spearman’s rank correlation coefficient. Pairs of IgG measures with a decline or rise were included as long as at least one time point was above LLOQ as well as restricted to the dataset where predicted levels below LLOQ were excluded. Where a high correlation was achieved and a low proportion of samples falling to below LLOQ and rising from below to above cut-offs, then samples taken at this time point could be used to back-calculate birth titres based on the decay rate. Statistical analyses were done with R studio (version 3.6.3).

## 3 Results

**Fig. 1:**
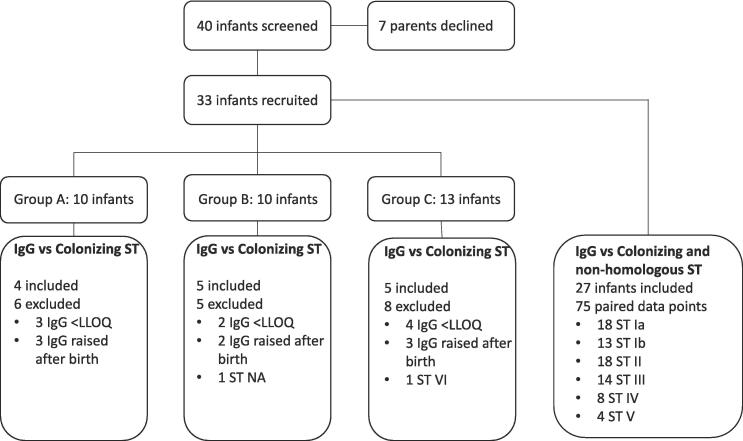
**Flow chart of available data and reasons for exclusions from half-life estimations.** GBS: Group B streptococcus; ST: Serotype; CPS: capsular polysaccharide; IgG: Immunoglobulin G; LLOQ: lower limit of quantification; NA: Not available.

The kinetics sub-study of the iGBS feasibility study enrolled 33 healthy infants born to mothers colonized with GBS who did not develop iGBS disease in the first three months of life (Fig. 1). Ten infants were enrolled in Group A, ten in Group B, and thirteen in Group C. Table 1 summarizes participants’ demographic and clinical characteristics. The median gestational age was 39 (range 35–42) weeks, with only two infants born < 37 weeks. The median maternal age was 34 years, with the youngest aged 22 and the oldest 43 years. Most infants were of white ethnicity (19; 57.6 %). Rectovaginal swabs were collected at a median gestation of 38 (range 35–42) weeks.

**Table 1:** Demographics and clinical characteristics of participating infants and their mothers.

	**Group A** n = 10	**Group B** n = 10	**Group C** n = 13	**Total** n = 33
**Maternal age, median (range)**	32 (26–43)	37 (22–43)	35 (27–43)	34 (22–43)

**Ethnicity, n (%)**				
Asian or Asian British	1 (10 %)	0 (0 %)	3 (23.1 %)	4 (12.1 %)
Black or Black British	0 (0 %)	1 (10 %)	2 (15.4 %)	3 (9.1 %)
White British	5 (50 %)	4 (40 %)	3 (23.1 %)	12 (36.4 %)
White Other	2 (20 %)	3 (30 %)	2 (15.4 %)	7 (21.2 %)
Other ethnic group	2 (20 %)	2 (20 %)	3 (23.1 %)	7 (21.2 %)

**Gravida, n (%)**				
1	5 (50 %)	8 (80 %)	6 (46.2 %)	19 (57.6 %)
2	2 (20 %)	0 (0 %)	0 (0 %)	2 (6.0 %)
3	0 (0 %)	2 (20 %)	3 (23.1 %)	5 (15.2 %)
4	0 (0 %)	0 (0 %)	4 (30.8 %)	4 (12.1 %)
>4	3 (30 %)	0 (0 %)	0 (0 %)	3 (9.1 %)

**Rupture of membranes, n (%)**				
>= 18 h	3 (30 %)	2 (20 %)	4 (30.8 %)	9 (28.1 %)

**Delivery type, n (%)**				
Vaginal	7 (70 %)	2 (20 %)	5 (38.5 %)	14 (42.4 %)
C-Section with rupture of membranes	1 (10 %)	4 (40 %)	3 (23.1 %)	8 (24.2 %)
C-Section without rupture of membranes	2 (20 %)	4 (40 %)	5 (38.5 %)	11 (33.3 %)
**Gestation at birth, median (range)**	39 (36–41)	39 (35–41)	39 (38–42)	39 (35–42)
**Gestation when swab collected, median (range)**	36 (35–39)	37 (35–41)	38 (35–42)	38 (35–42)

**Infant sex, n (%)**				
Female	5 (50 %)	3 (30 %)	8 (61.5 %)	16 (48.5 %)

**Intrapartum antibiotics, n (%)**				
Yes	6 (60 %)	8 (80 %)	10 (76.9 %)	24 (72.7 %)

**Serotype Distribution**				
Ia	3 (30 %)	2 (20 %)	4 (30.8 %)	9 (28.1 %)
Ib	0 (0 %)	1 (10 %)	3 (23.1 %)	4 (12.5 %)
II	1 (10 %)	2 (20 %)	3 (23.1 %)	6 (18.8 %)
III	5 (50 %)	2 (20 %)	2 (15.4 %)	9 (28.1 %)
IV	0 (0 %)	1 (10 %)	0 (0 %)	1 (3.1 %)
V	1 (10 %)	1 (10 %)	0 (0 %)	2 (6.2 %)
VI	0 (0 %)	0 (0 %)	1 (7.7 %)	1 (3.1 %)
NA	0 (0 %)	1 (0 %)	0 (0 %)	1 (3.1 %)

Group A: Blood collection day 21–35, Group B: Blood collection day 49–63, Group C: Blood collection day 77–91

**Fig. 2:**
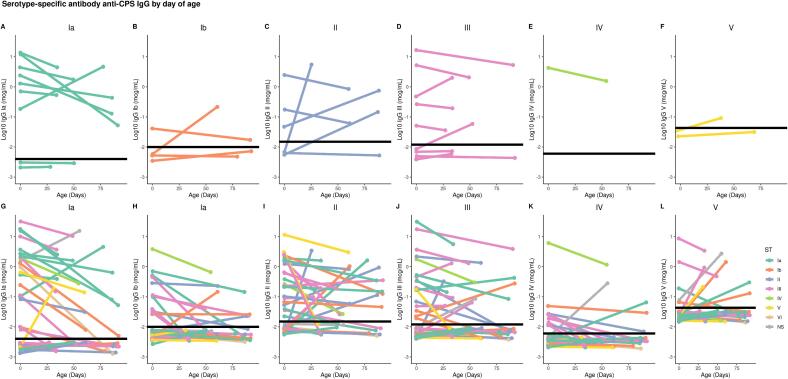
**Serotype-specific anti-GBS CPS IgG (expressed as log10) by day of age.** (A) Homologous IgG responses to ST Ia. (B) Homologous IgG responses to ST Ib. (C) Homologous IgG responses to ST II. (D) Homologous IgG responses to ST III. (E) Homologous IgG responses to ST IV. (F) Homologous IgG responses to ST V. (G) Homologous and non-homologous IgG responses to ST Ia. (H) Homologous and non-homologous IgG responses to ST Ib. (I) Homologous and non-homologous IgG responses to ST II. (J) Homologous and non-homologous IgG responses to ST III. (K) Homologous and non-homologous IgG responses to ST IV. (L) Homologous and non-homologous IgG responses to ST V. Colors depict the colonizing ST. Black line shows the LLOQ for each serotype. Values below LLOQ were plotted as LLOQ/2. Values below LLOQ were jittered to avoid overplotting. GBS: Group B streptococcus; ST: Serotype; CPS: capsular polysaccharide; IgG: Immunoglobulin G; LLOQ: lower limit of quantification.

**Fig. 3:**
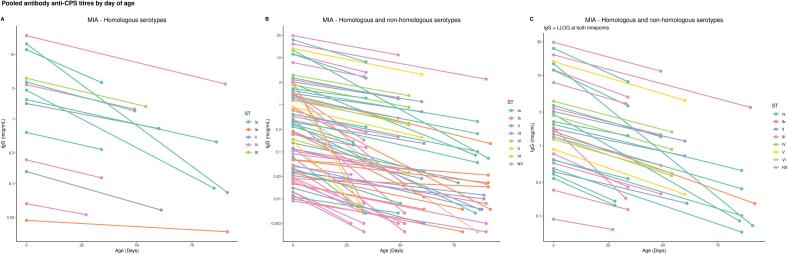
**Pooled antibody anti-CPS titres by day of age.** (A) Pooled placentally transferred anti-GBS CPS IgG by day of age against colonizing serotype for paired samples with decline of antibodies over time. (B) Pooled placentally transferred anti-GBS CPS IgG by day of age for colonizing and non-homologous serotypes for paired samples with decline of antibodies over time. (C) Pooled placentally transferred anti-GBS CPS IgG by day of age for colonizing and non-homologous serotypes for paired samples with decline of antibodies over time and IgG > LLOQ at both time points. Colors depict the colonizing ST. GBS: Group B streptococcus; ST: Serotype; CPS: capsular polysaccharide; IgG: Immunoglobulin G.

The serotype-specific anti-GBS CPS IgG concentrations in cord and infant blood against the colonizing serotype were plotted for each infant (Fig. 2**A-F**). There were nine (27.2 %) paired samples of cord and infant blood with both IgG values below LLOQ. In eight (24.2 %) participants, IgG values increased between birth and the time of infant blood collection, five of whom had birth levels below the LLOQ. After these data were excluded, 14 paired samples were used to calculate the antibody decay rate against the colonizing serotype. Of these 14 samples, six infants were exposed to ST Ia (42.9 %), four to ST III (28.6 %), two to ST II (14.3 %), one to ST Ib (7.1 %), and one to ST IV (7.1 %). The half-life of transplacental maternal IgG antibodies was 27.9 (95 % CI: 19.9–46.2) days (Fig. 3**A**).

**Fig. 4:**
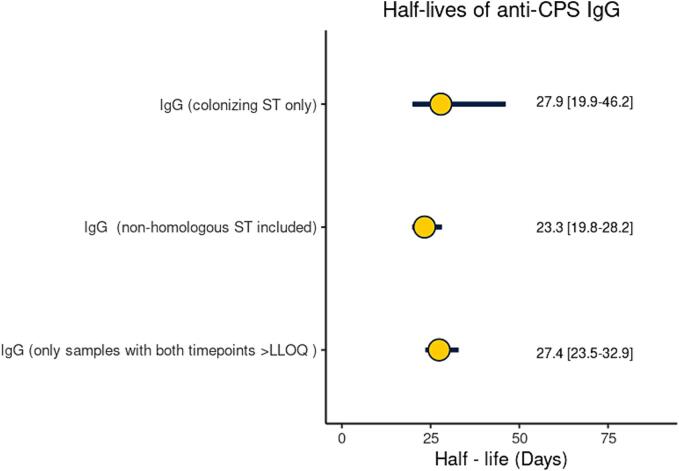
**Half-lives of transplacental antibody with point estimate and 95 % CI based on IgG against colonising serotype, against colonising and non-homologous serotypes, against colonising and non-homologous serotypes only when IgG > LLOQ at both time points.** GBS: Group B streptococcus; ST: Serotype; CPS: capsular polysaccharide; IgG: Immunoglobulin G; CI: confidence interval.

**Supplementary Fig. 1:**
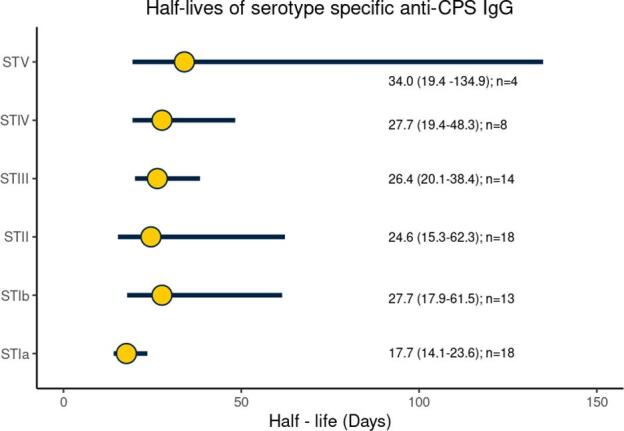
Half-lives of serotype-specific transplacental antibody with point estimate and 95% CI based on IgG against colonizing and non-homologous serotypes. GBS: Group B streptococcus; ST: Serotype; CPS: capsular polysaccharide; IgG: Immunoglobulin G; CI: confidence interval.

When IgG against non-homologous CPS was included, 76 pairs of IgG measures from 30 infants were used to estimate the half-life of IgG (Fig. 1). There were 95 (47.9 %) paired cord and infant blood samples with both values below LLOQ (+20.7 % vs colonizing ST analysis; p = 0.04), and 27 (13.6 %) pairs with raised IgG measures after birth (-10.6 % vs colonizing ST analysis; p = 0.19). The individual slopes for all paired data points are shown in Fig. 2**G-L**. Half-lives were 17.7 (14.1–23.6), 27.7 (17.9–61.5), 24.6 (15.3–62.3), 26.4 (20.1–38.4), 27.7 (19.4–48.3) and 34.0 (19.4––134.9) days for ST Ia, ST Ib, ST II, ST III, ST IV, ST V, respectively (Fig. S1). When all paired results were pooled together, the half-life of IgG was 23.3 (19.8–28.2) days (Fig. 3**B**). In the sensitivity analysis, the estimation of IgG half-life was limited to 34 paired data points with both time points > LLOQ and no rise after birth. The half-life was 27.4 [23.5–32.9] days (Fig. 3**C**). A Cleveland plot summarizing the results of the primary and sensitivity analyses is shown in Fig. 4.

The decay rate of antibodies did not vary by maternal age (p = 0.7), ethnicity (p = 0.1), gravidity (p = 0.1), gestational age (p = 0.7) or infant sex (p = 0.1).

**Fig. 5:**
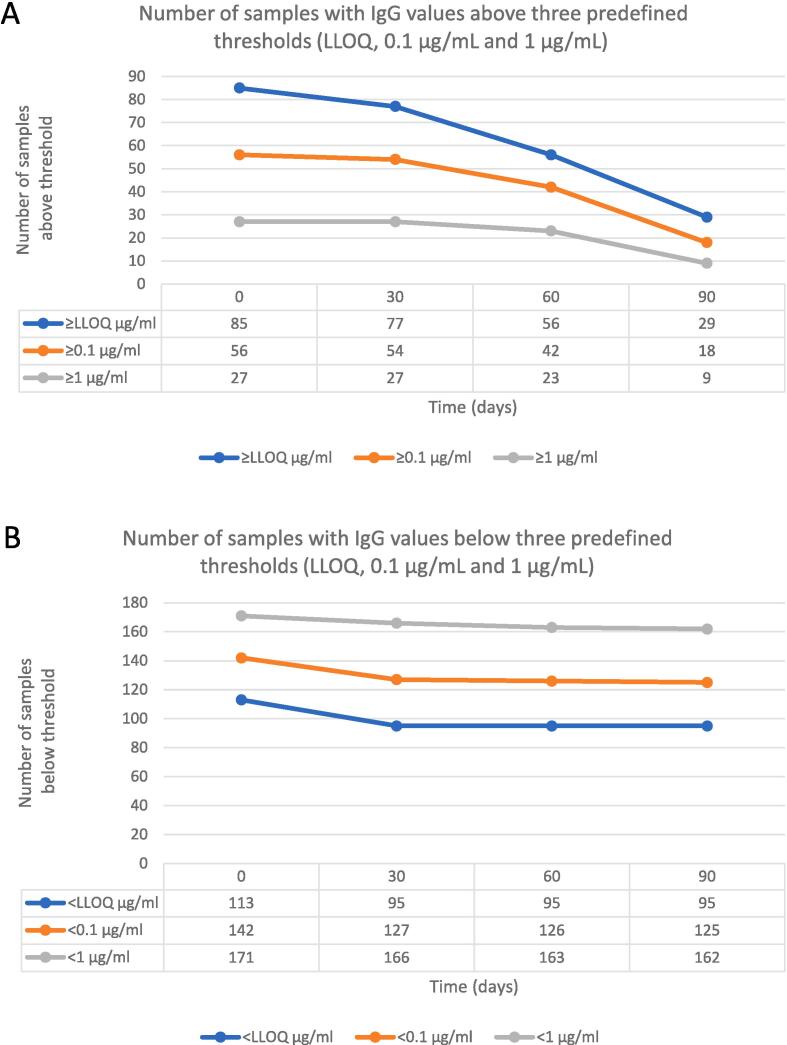
**Prediction of samples with IgG values below or above predefined thresholds.** (A) Number of samples with IgG values below three predefined thresholds (LLOQ, 0.1 μg/mL and 1 μg/mL) at birth that were predicted to remain below each threshold at three time points (day 30, 60, 90 of age) (B) Number of samples with IgG values above three predefined thresholds (LLOQ, 0.1 μg/mL and 1 μg/mL) at birth that were predicted to remain above each threshold at three time points (day 30, 60, 90 of age).

**Fig. 6:**
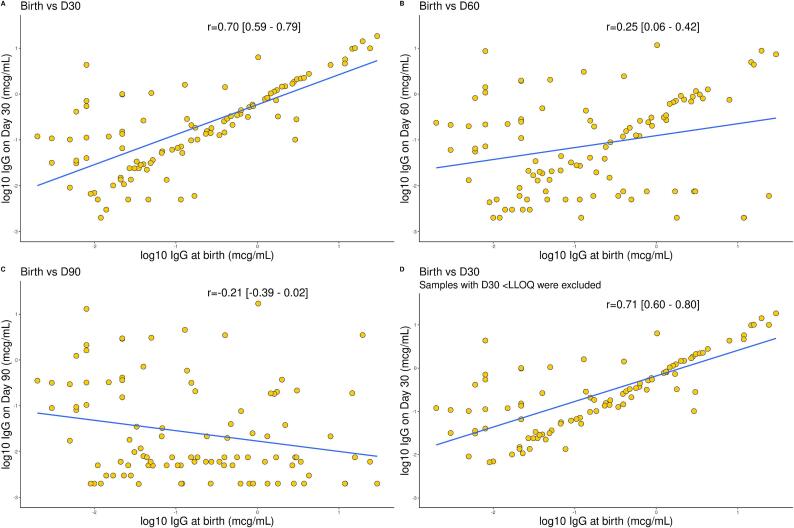
**Comparison of log10 placentally transferred anti-GBS CPS IgG at birth and day 30, 60 and 90 of age** (A) Comparison of log10 placentally transferred anti-GBS CPS IgG at birth and day 30 of age for the homologous and non-homologous ST. (B) Comparison of log10 placentally transferred anti-GBS CPS IgG at birth and day 60 of age for the homologous and non-homologous ST. (C) Comparison of log10 placentally transferred anti-GBS CPS IgG at birth and day 90 of age for the homologous and non-homologous ST. (D) Comparison of log10 placentally transferred anti-GBS CPS IgG at birth and day 30 of age for the homologous and non-homologous ST, after exclusion of samples with a value < LLOQ on day 30. Statistical significance assessed by Spearman correlation. X axis scale differs among plots. GBS: Group B streptococcus; CPS: capsular polysaccharide; IgG: Immunoglobulin G; LLOQ: lower limit of quantification.

In the correlation analysis, only a small proportion (<10 %) of samples with detectable antibodies at birth had predicted titres below LLOQ on day 30. In contrast, approximately a third of samples were predicted to fall below < LLOQ on day 60, and two-thirds below < LLOQ on day 90 (Fig. 5**A**). Similarly, over 95 % of samples with birth titres of ≥ 0.1 mcg/ml and ≥ 1 mcg/ml, were predicted to have detectable values on day 30, 75–85 % on day 60 and only around 33 % on day 90 (Fig. 5**A**). Notably, predicted titres raised above the LLOQ in approximately one-sixth of samples with undetectable birth titres, a small but not insignificant proportion (Fig. 5**B**). A strong correlation was observed between IgG concentrations at birth and day 30 (r = 0.70 [95 % CI: 0.59–0.79]), but not day 60 (r = 0.25 [0.06–0.42]) and day 90 (r = -0.21 [-0.39–0.02]) (Fig. 6**A-C**). When the analysis was restricted to day 30 and predicted levels below LLOQ were excluded, the correlation coefficient was 0.71 (0.60–0.80) (Fig. 6**D**).

## 4 Discussion

This study provides the first estimates of the half-life of placentally transferred anti-GBS CPS IgG concentrations in healthy infants following natural exposure to GBS. The half-life of IgG against the colonizing serotype was 27.9 (95 % CI: 19.9–46.2) days. When IgG against non-homologous CPS was added, the half-life was 23.3 (19.8–28.2) days. Including IgG against non-homologous serotypes allowed for a larger number of samples to be included and showed equivalent results to the colonizing serotype analysis with narrower confidence intervals. The presence of antibodies against the non-colonizing serotype probably reflects previous colonization episodes due to different serotypes. GBS colonization changes over time, but serotype-specific anti-GBS CPS antibodies tend to remain stable [30]. Another possible explanation is co-colonization with more than one serotype [31]. The prevalence of multiple serotype carriage is probably under-recognised, as it requires laboratory techniques that allow for multiple colony selection [32]. When the analysis of IgG against homologous and non-homologous serotypes was restricted to paired data points with both time points above LLOQ, the half-life was 27.4 (23.5–32.9) days. Since the exact time IgG fell below LLOQ is unknown, the estimated half-life from paired samples that fall below the LLOQ might be less accurate, introducing a bias to the analysis. In this sense, the estimate of 27.4 days from the sensitivity analysis provides a better estimate of the antibody decay and could be used to back-calculate birth titres from an infant sample that is above the LLOQ. Given that half of LOD cases present in the first month of life and three quarters by six weeks of age [3], it is important that antibodies are still detectable at 30 days.

The point estimates in our naturally-acquired antibody study were comparable but shorter than the previously reported half-life of vaccine-derived anti-GBS CPS IgG by a magnitude of one to two weeks. A double-blind, placebo-controlled trial of a monovalent GBS CPS ST III - tetanus toxoid conjugate vaccine reported a half-life of ST III specific IgG of 35 days [33], [34]. Half-lives of IgG against ST Ia, Ib, and III following immunization with a trivalent non-adjuvanted CRM_197_-conjugated GBS vaccine were 39–46 days, depending on the ST [35]. However, corresponding confidence intervals were not provided in the GBS vaccine studies above; therefore, it is unknown whether these differences are statistically significant. In addition, the calculations used to derive these estimates are not described in detail; therefore it is unclear whether there are any methodological differences that would account for these differences. Similar estimates to those seen with GBS have been reported for half-lives of other antigen-specific antibodies following maternal vaccination. A recent *meta*-analysis of placentally transferred antibodies against diphtheria toxoid, tetanus toxoid, fimbriae, pertussis toxoid, filamentous haemagglutinin, and pertactin antigens reported half-lives between 29 and 35 days [29]. A previous study of trivalent inactivated influenza vaccine in pregnancy showed half-lives of 45, 44, and 43 days against H1N1, H3N2, and B/Victoria strains, respectively [36].

Less is known about the kinetics of passively acquired antibodies in infants secondary to natural exposure. Pawlowski et al. estimated the half-life of naturally acquired IgG against the N-terminal domains of the GBS alpha-like proteins (Alp) *a*C and Rib in paired cord and infant blood samples one month after delivery to be 40 and 38 days, respectively, although no confidence intervals were reported [37]. In studies of pneumococcal and meningococcal A and C antibodies, the half-lives were 43, 43, and 40 days [28]. The half-life of neutralizing antibodies against respiratory syncytial virus was 38 days in a study of 149 asymptomatic mother-infant pairs in Bangladesh [38]. These differences could be attributed to different target antigens and assays or reflect differences between natural immunity and vaccine-induced immunity, for example in IgG subclass distribution.

In our cohort of healthy infants born > 35 weeks gestation, we did not find any factors that affected the decay rate of placentally transferred anti-GBS CPS IgG. Although our results might have been affected by the small sample size, they are in line with those of previous studies [28], [29]. Of note, preterm birth is associated with a lower concentration of placentally transferred maternal IgG at birth due to differences in the expression of the FcRn receptor [15], but this does not necessarily impact decay rates after birth [28], [29].

Several GBS vaccine candidates are currently in development [39]. Due to the complexities of a vaccine efficacy trial with a clinical primary endpoint, a serocorrelate of protection against iGBS disease could facilitate the licensure of such a vaccine [20]. This would be derived from studies of natural immunity estimating antibody concentrations associated with protection against iGBS disease. The consensus within the scientific community is that infant serum is the preferable source for IgG measurement for this purpose rather than maternal serum that might not reflect the infant antibody concentrations at birth due to variations in placental antibody transfer ratios [40]. However, measuring cord blood IgG from a large number of infants in seroepidemiological studies can be challenging from a practical point of view. In this study, the correlation analysis indicated that it is possible to use infant sera with IgG titres over LLOQ that were collected up to 30 days after birth to predict titres at birth using the estimated half-life from the sensitivity analysis. Back-calculating was less accurate beyond day 30, since a significant proportion of samples declined below LLOQ on day 60 and especially day 90, even when started with a fairly high level at birth. Of note, we found a significant proportion of participants showing rising IgG levels after birth. A possible explanation is that mother-to-infant transmission of GBS may occur after delivery. Based on studies in Italy, France, the Gambia, and Japan, between a fifth and a quarter of infants become colonised with the same strain as their mothers after hospital discharge, despite adequate IAP and negative GBS screening at birth [41], [42], [43], [44]. In studies with juvenile mice, intestinal colonisation with GBS induces an endogenous IgG response within 20 days of exposure [45]. Ongoing exposure to GBS after birth could potentially induce the production of antibodies in human infants, but the evidence for this is lacking [46]. Although not statistically significant, the lower proportion of samples with an unexpected rise against the non-colonizing compared to the colonizing serotype in our study supports this hypothesis, as it is biologically plausible that the antibodies produced secondary to postnatal exposure will target the colonizing serotype. In any case, these findings from healthy infants exposed to GBS need to be validated in actual cases of infant disease. A large UK seroepidemiological study (iGBS3) currently underway aims to determine the correlation between cord and acute phase IgG in infants with iGBS disease [47]. If half-lives in cases and controls look similar, it might be possible to combine these data with cases to increase power for the back-prediction. In addition, our observations suggest that maternal age, ethnicity, gravidity, gestational age and infant sex do not modify antibody decline rates between birth and three months of life in healthy infants. These findings could indicate that any differences in the half-lives in cases compared to controls might be the result of the disease process (consumption or convalescence) rather than confounded by maternal or infant factors.

Our study has some limitations. First, we recruited a relatively small number of participants. Recruitment was affected by the restrictions implemented due to the COVID-19 pandemic, which led to the early closure of the study. The imbalance in the size of the three groups reflects the methodological limitations of simple randomization when the number of participants is small. We partially mitigated the problem of the small number of participants by including IgG against non-homologous CPS that allowed for analysis of a larger dataset. Whereas the primary analysis included 14 paired data points, the secondary analyses included 75 paired data points (Fig. 1), thus resulting in narrower confidence intervals (Fig. 4). The fact that the point estimates of IgG half-lives only differed for a few days (<5 days) between the primary and secondary analyses gives us confidence in our approach. However, we could not estimate a serotype-specific decay rate with meaningful corresponding confidence intervals. It is plausible that differences might exist between serotypes or capsular groups, even if these amount to differences of only a few days [28], [29]. Second, the generalisability of our findings might be limited by the fact that all infants in our study were born after 35 weeks of gestation and our cohort predominantly consisted of white ethnic groups from a single country. However, a previous systematic review found that antibody decay rates did not differ by infant gestational age or World Bank income categories for non-CPS antigens [29]. Third, IgG was measured at two time points for each participant. A third timepoint might have contributed to a better understanding of the antibody kinetics, although it is unlikely that a linear decline between birth and three months of age would have changed. Given the practical difficulties and the low parental acceptability of repeated venepunctures in healthy non-hospitalized infants, innovative and less invasive approaches like dried blood spots could be tested in the future [48]. Fourth, we have not collected data on infant or maternal colonization after birth. Paired maternal and infant swabs and breast milk might have allowed us to understand the dynamics of GBS colonization and IgG consistency of breast milk in conjunction with the infant antibody kinetics and inform hypotheses about their interplay. Fifth, a high number of samples had IgG values below LLOQ [41]. Starting with lower IgG values at birth meant that many samples were below the LLOQ at the second time point. Since the exact time IgG fell below LLOQ is unknown, the estimated half-life for these paired samples is possibly overestimated. We mitigated this issue by performing a sensitivity analysis including only samples with both time points above LLOQ. However, a large proportion of our paired samples had both time points below LLOQ and were therefore excluded from the analyses.

## 5 Conclusion

Our results provide a basis for future investigations into the use of antibody kinetics in defining a serocorrelate of protection against late-onset iGBS disease. In combination with clinical trials, such an approach could facilitate the licensure of a GBS vaccine to prevent both early and late-onset iGBS disease.

Funding

The iGBS feasibility study was funded by the National Institute for Health Research Health Technology Assessment programme as project number 17/153/01. This work was also supported by a small internal grant from St George’s, University of London, Institute for Infection & Immunity Research Funding Scheme 2020 to cover consumables and travel costs.

## CRediT authorship contribution statement

**Konstantinos Karampatsas:** Data curation, Formal analysis, Funding acquisition, Investigation, Writing – original draft, Writing – review & editing. **Tom Hall:** Methodology, Writing – review & editing. **Merryn Voysey:** Supervision, Writing – review & editing. **Clara Carreras-Abad:** Data curation, Investigation, Writing – review & editing. **Madeleine Cochet:** Project administration, Resources, Writing – review & editing. **Laxmee Ramkhelawon:** Methodology, Writing – review & editing. **Elisabeth Peregrine:** Investigation, Writing – review & editing. **Nick Andrews:** Methodology, Supervision, Writing – review & editing. **Paul T. Heath:** . **Kirsty Le Doare:** Conceptualization, Funding acquisition, Supervision, Writing – review & editing.

## Declaration of competing interest

The authors declare the following financial interests/personal relationships which may be considered as potential competing interests: [Paul Heath reports financial support was provided by National Institute for Health Research. MV is a contributor to intellectual property licensed by Oxford University Innovation to AstraZeneca. PTH has conducted studies on behalf of St George’s University of London funded by GBS vaccine manufacturers, including Minervax and Pfizer, but receives no personal funding for these activities. KLD is supported by Future Leaders Fellowships by UK Research and Innovation Future Leaders Fellowship (MR/S016570/1) and has conducted studies on behalf of St George’s University of London funded by GBS vaccine manufacturers, including Minervax and Pfizer, but receives no personal funding for these activities. The authors declare that they have no known competing financial interests or personal relationships that could have appeared to influence the work reported in this paper. If there are other authors, they declare that they have no known competing financial interests or personal relationships that could have appeared to influence the work reported in this paper.].

## Data Availability

Data will be made available on request.

## References

[1] Gonçalves B.P., Procter S.R., Paul P., Chandna J., Lewin A., Seedat F. (2022). Group B streptococcus infection during pregnancy and infancy: estimates of regional and global burden. Lancet Glob Health.

[2] Horváth-Puhó E., van Kassel M.N., Gonçalves B.P., de Gier B., Procter S.R., Paul P. (2021). Mortality, neurodevelopmental impairments, and economic outcomes after invasive group B streptococcal disease in early infancy in Denmark and the Netherlands: a national matched cohort study. Lancet Child Adolesc Health.

[3] Nanduri S.A., Petit S., Smelser C., Apostol M., Alden N.B., Harrison L.H. (2019). Epidemiology of invasive early-onset and late-onset group b streptococcal disease in the United States, 2006 to 2015: multistate laboratory and population-based surveillance. JAMA Pediatr.

[4] Hall J., Adams N.H., Bartlett L., Seale A.C., Lamagni T., Bianchi-Jassir F. (2017). Maternal disease with group B streptococcus and serotype distribution worldwide: systematic review and meta-analyses. Clin Infect Dis.

[5] Seale A.C., Blencowe H., Bianchi-Jassir F., Embleton N., Bassat Q., Ordi J. (2017). Stillbirth with group B streptococcus disease worldwide: systematic review and meta-analyses. Clin Infect Dis.

[6] Dangor Z., Kwatra G., Izu A., Lala S.G., Madhi S.A. (2014). Review on the association of group B streptococcus capsular antibody and protection against invasive disease in infants. Expert Rev Vacc.

[7] Vekemans J., Moorthy V., Friede M., Alderson M.R., Sobanjo-Ter Meulen A., Baker C.J. (2019). Maternal immunization against Group B streptococcus: World Health Organization research and development technological roadmap and preferred product characteristics. Vaccine.

[8] Baker C.J., Kasper D.L. (1976). Correlation of maternal antibody deficiency with susceptibility to neonatal group B streptococcal infection. N Engl J Med.

[9] Lin F.C., Weisman L.E., Azimi P.H., Philips J.B., Clark P., Regan J. (2004). Level of maternal IgG anti-group B streptococcus type III antibody correlated with protection of neonates against early-onset disease caused by this pathogen. J Infect Dis.

[10] Dangor Z., Kwatra G., Izu A., Adrian P., Cutland C.L., Velaphi S. (2015). Correlates of protection of serotype-specific capsular antibody and invasive group B streptococcus disease in South African infants. Vaccine.

[11] Madhi S.A., Izu A., Kwatra G., Jones S., Dangor Z., Wadula J. (2021). Association of group B streptococcus (GBS) serum serotype-specific anticapsular immunoglobulin G concentration and risk reduction for invasive GBS disease in South African infants: an observational birth-cohort. Matched Case-Control Study Clin Infec Dis.

[12] Fabbrini M., Rigat F., Rinaudo C.D., Passalaqua I., Khacheh S., Creti R. (2016). The protective value of maternal group B streptococcus antibodies: quantitative and functional analysis of naturally acquired responses to capsular polysaccharides and pilus proteins in european maternal sera. Clin Infect Dis.

[13] Calvert A., Jones C.E. (2017). Placental transfer of antibody and its relationship to vaccination in pregnancy. Curr Opin Infect Dis.

[14] Malek A., Sager R., Kuhn P., Nicolaides K.H., Schneider H. (1996). Evolution of maternofetal transport of immunoglobulins during human pregnancy. Am J Reprod Immunol.

[15] Lozano N.A., Lozano A., Marini V., Saranz R.J., Blumberg R.S., Baker K. (2018). Expression of FcRn receptor in placental tissue and its relationship with IgG levels in term and pre-term newborns. Am J Reprod Immunol.

[16] Albrecht M., Arck P.C. (2020). Vertically transferred immunity in neonates: mothers. Mecha Mediators Front Immunol.

[17] Clements T., Rice T.F., Vamvakas G., Barnett S., Barnes M., Donaldson B. (2020). Update on transplacental transfer of IgG subclasses: impact of maternal and fetal factors. Front Immunol.

[18] Marchant A., Sadarangani M., Garand M., Dauby N., Verhasselt V., Pereira L. (2017). Maternal immunisation: collaborating with mother nature. Lancet Infect Dis.

[19] Jennewein M.F., Goldfarb I., Dolatshahi S., Cosgrove C., Noelette F.J., Krykbaeva M. (2019). Fc glycan-mediated regulation of placental antibody transfer. Cell.

[20] Le Doare K., Kampmann B., Vekemans J., Heath P.T., Goldblatt D., Nahm M.H. (2019). Serocorrelates of protection against infant group B streptococcus disease. Lancet Infect Dis.

[21] Carreras-Abad C., Cochet M., Hall T., Ramkhelawon L., Khalil A., Peregrine E. (2019). Developing a serocorrelate of protection against invasive group B streptococcus disease in pregnant women: a feasibility study. Health Technol Assess (Rockv).

[22] SMI B 58: detection of carriage of group B streptococci - GOV.UK n.d. https://www.gov.uk/government/publications/smi-b-58-processing-swabs-for-group-b-streptococcal-carriage (accessed February 2, 2023).

[23] Imperi M., Pataracchia M., Alfarone G., Baldassarri L., Orefici G., Creti R. (2010). A multiplex PCR assay for the direct identification of the capsular type (Ia to IX) of Streptococcus agalactiae. J Microbiol Methods.

[24] To K.N., Cornwell E., Daniel R., Goonesekera S., Jauneikaite E., Chalker V. (2019). Evaluation of matrix-assisted laser desorption ionisation time-of-flight mass spectrometry (MALDI-TOF MS) for the Identification of group B streptococcus. BMC Res Notes.

[25] Buurman E.T., Timofeyeva Y., Gu J., Kim J.H., Kodali S., Liu Y. (2019). A novel hexavalent capsular polysaccharide conjugate vaccine (GBS6) for the prevention of neonatal group B streptococcal infections by maternal immunization. J Infect Dis.

[26] Esadze A., Grube C.D., Wellnitz S., Singh S., Nguyen H.H., Gaylord M.A. (2023). Calibration of a serum reference standard for Group B streptococcal polysaccharide conjugate vaccine development using surface plasmon resonance. Npj Vaccines.

[27] Madhi S.A., Anderson A.S., Absalon J., Radley D., Simon R., Jongihlati B. (2023). Potential for maternally administered vaccine for infant group B streptococcus. N Engl J Med.

[28] Voysey M., Pollard A.J., Sadarangani M., Fanshawe T.R. (2017). Prevalence and decay of maternal pneumococcal and meningococcal antibodies: a meta-analysis of type-specific decay rates. Vaccine.

[29] Oguti B., Ali A., Andrews N., Barug D., Anh Dang D., Halperin S.A. (2022). The half-life of maternal transplacental antibodies against diphtheria, tetanus, and pertussis in infants: an individual participant data meta-analysis. Vaccine.

[30] Haeusler I.L., Daniel O., Isitt C., Watts R., Cantrell L., Feng S. (2022). Group B streptococcus (GBS) colonization is dynamic over time, whilst GBS capsular polysaccharides-specific antibody remains stable graphical abstract. Clin Exp Immunol.

[31] Khatami A., Randis T.M., Tavares L., Gegick M., Suzman E., Ratner A.J. (2019). Vaginal co-colonization with multiple group B streptococcus serotypes. Vaccine.

[32] Barro C., Salloum M., Lim S., Delputte P., Le Doare K. (2023). Simultaneous carriage of multiple serotypes of group B streptococcus: systematic review and meta-analysis. Vaccine.

[33] Baker C.J., Rench M.A., McInnes P. (2003). Immunization of pregnant women with group B streptococcal type III capsular polysaccharide-tetanus toxoid conjugate vaccine. Vaccine.

[34] Edwards M.S., Rench M.A., Baker C.J. (2015). Relevance of age at diagnosis to prevention of late-onset group B streptococcal disease by maternal immunization. Pediatr Infect Dis J.

[35] Madhi S.A., Koen A., Cutland C.L., Jose L., Govender N., Wittke F. (2017). Antibody kinetics and response to routine vaccinations in infants born to women who received an investigational trivalent group B streptococcus polysaccharide CRM197-conjugate vaccine during pregnancy. Clin Infect Dis.

[36] Nunes M.C., Cutland C.L., Dighero B., Bate J., Jones S., Hugo A. (2015). Kinetics of hemagglutination-inhibiting antibodies following maternal influenza vaccination among mothers with and those without HIV infection and their infants. J Infect Dis.

[37] Pawlowski A., Lannergård J., Gonzalez-Miro M., Cao D., Larsson S., Persson J.J. (2022). A group B streptococcus alpha-like protein subunit vaccine induces functionally active antibodies in humans targeting homotypic and heterotypic strains. Cell Rep Med.

[38] Chu H.Y., Steinhoff M.C., Magaret A., Zaman K., Roy E., Langdon G. (2014). Respiratory syncytial virus transplacental antibody transfer and kinetics in mother-infant pairs in Bangladesh. J Infect Dis.

[39] Carreras-Abad C., Ramkhelawon L., Heath P.T., Le Doare K. (2020). A vaccine against group B streptococcus: recent advances. Infect Drug Resist.

[40] Gilbert P.B., Isbrucker R., Andrews N., Goldblatt D., Heath P.T., Izu A. (2022). Methodology for a correlate of protection for group B streptococcus: report from the bill & melinda gates foundation workshop held on 10 and 11 february 2021. Vaccine.

[41] Berardi A., Rossi C., Creti R., China M., Gherardi G., Venturelli C. (2013). Group B streptococcal colonization in 160 mother-baby Pairs: a prospective cohort study. J Pediatr.

[42] Le Doare K., Jarju S., Darboe S., Warburton F., Gorringe A., Heath P.T. (2016). Risk factors for group B streptococcus colonisation and disease in Gambian women and their infants. J Infect.

[43] Toyofuku M., Morozumi M., Hida M., Satoh Y., Sakata H., Shiro H. (2017). Effects of intrapartum antibiotic prophylaxis on neonatal acquisition of group B streptococci. J Pediatr.

[44] Tazi A., Plainvert C.C., Anselem O., Ballon M., Marcou V.V., Seco A.A. (2019). Risk factors for infant colonization by hypervirulent CC17 group B streptococcus: toward the understanding of late-onset disease. Clin Infect Dis.

[45] Vaz M.J., Purrier S.A., Bonakdar M., Chamby A.B., Ratner A.J., Randis T.M. (2021). The impact of circulating antibody on group b streptococcus intestinal colonization and invasive disease. Infect Immun.

[46] Semmes E.C., Chen J.L., Goswami R., Burt T.D., Permar S.R., Fouda G.G. (2021). Understanding early-life adaptive immunity to guide interventions for pediatric health. Front Immunol.

[47] Study Details | Development of a Serocorrelate of Protection Against Invasive Group B Streptococcus Disease | ClinicalTrials.gov n.d. https://clinicaltrials.gov/study/NCT04735419 (accessed November 21, 2023).

[48] E. Auma T. Hall S. Chopra S. Bilton L. Ramkhelawon F. Amini et al. Using Dried Blood Spots for a Sero-Surveillance Study of Maternally Derived Antibody against Group B Streptococcus 2023 10.3390/vaccines11020357. 10.3390/vaccines11020357 PMC9966576 36851236

